# Hepatosplenic Candidiasis Detected by ^18^F-FDG-PET/CT

**DOI:** 10.7508/aojnmb.2016.02.007

**Published:** 2016

**Authors:** Domenico Albano, Giovanni Bosio, Mattia Bertoli, Giulia Petrilli, Francesco Bertagna

**Affiliations:** 1 Nuclear Medicine, Spedali Civili di Brescia, Brescia, Italy; 2 Department of Molecular and Translational Medicine, Anatomic Pathology Section, Spedali Civili di Brescia, Brescia, Italy

**Keywords:** ^18^F-FDG-PET/CT, Acute myeloid leukemia, Hepatosplenic candidiasis

## Abstract

Hepatosplenic candidiasis is a fungal infection, which mostly affects patients with hematologic malignancies such as leukemia. The pathogenesis of this infection is not clear yet, and the liver is the most commonly affected organ. Diagnosis of hepatosplenic candidiasis can be only established via biopsy, since computed tomography (CT) scan, ultrasonography, and magnetic resonance imaging (MRI) yield non-specific results. The role of fluorine-18 fluorodeoxyglucose positron emission tomography /computed tomography (^18^F-FDG PET/CT) in diagnosis of hepatosplenic candidiasis remains undetermined, considering a few evidences in the literature. In this case report, we present the case of a 47-year-old patient, affected by acute myeloid leukemia, which was treated with three cycles of chemotherapy, resulting in the development of neutropenia and fever following the last cycle. The ^18^F-FDG PET/CT scan showed some foci of intense FDG uptake in the liver and spleen. The subsequent diagnostic investigations (i.e., abdominal CT scan and biopsy) were suggestive of hepatosplenic candidiasis. The patient was started on antifungal treatment with fluconazole. After one month, the clinical conditions were resolved, and the subsequent abdominal CT scan was negative.

## Introduction

Hepatosplenic candidiasis or chronic disseminated candidiasis is an invasive fungal infection, affecting neutropenic patients. Hepatosplenic candidiasis occurs almost exclusively in patients with acute leukemia and is rarely associated with other conditions (1, 2). Although the pathogenesis of this infection is not well understood, a host inflammatory response is hypothesized to play a crucial role in this condition (2).

Fever is the most common sign of hepatosplenic candidiasis, often accompanied by right upper quadrant pain, nausea, vomiting, and anorexia (3). A definitive diagnosis can be only established via biopsy, which can reveal multiple granulomas with specific stains, yeasts, and hyphal forms (1).

In this report, we present the case of a patient, affected by acute myeloid leukemia, who developed hepatosplenic candidiasis following chemotherapy. ^18^FDG-PET/CT scan was shown to be useful in detecting hepatosplenic candidiasis and the subsequent patient management. In the literature, only few reported cases have shown FDG uptake in hepatosplenic candidiasis, and the utility of fluorodeoxyglucose (FDG) PET/CT scan is still under debate (7-9).

## Case report

A 47-year-old woman, affected by acute myeloid leukemia, was treated with three cycles of chemotherapy (using prednisone and vincristine). During the final cycle of chemotherapy, she developed neutropenia and fever, which were non-responsive to antibacterial therapy,

The patient underwent ^18^F-FDG PET/CT scan to determine the cause of persistent fever. PET/CT scan was acquired 60 min after the intravenous injection of 217.02 MBq of ^18^F-FDG (3.5 MBq/kg) on Discovery 690 tomograph (General Electric Company, Milwaukee, WI, USA; 64-slice CT scan, 80 mA, 120 kV; 2.5 min/bed; 256×256 matrix, 60 cm field of view).

**Figure 1:**
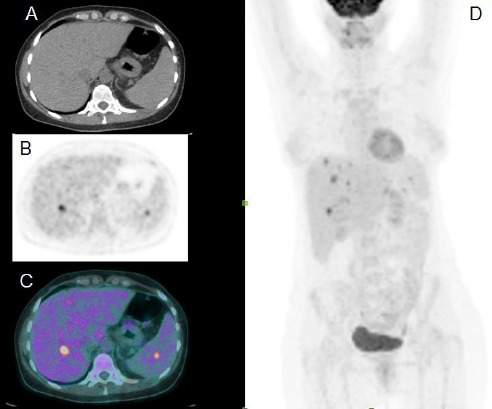
Axial CT (A), axial PET (B), and axial ^18^F-FDG PET/CT (C) images, showing increased FDG uptake in one hepatic (segment VI, SUV_max_=7.9) and one splenic (SUV_max_=5.2) lesion. Maximum intensity projection (MIP) image of the body (D) shows multiple areas of FDG uptake in the liver, with no other pathological uptakes

**Figure 2:**
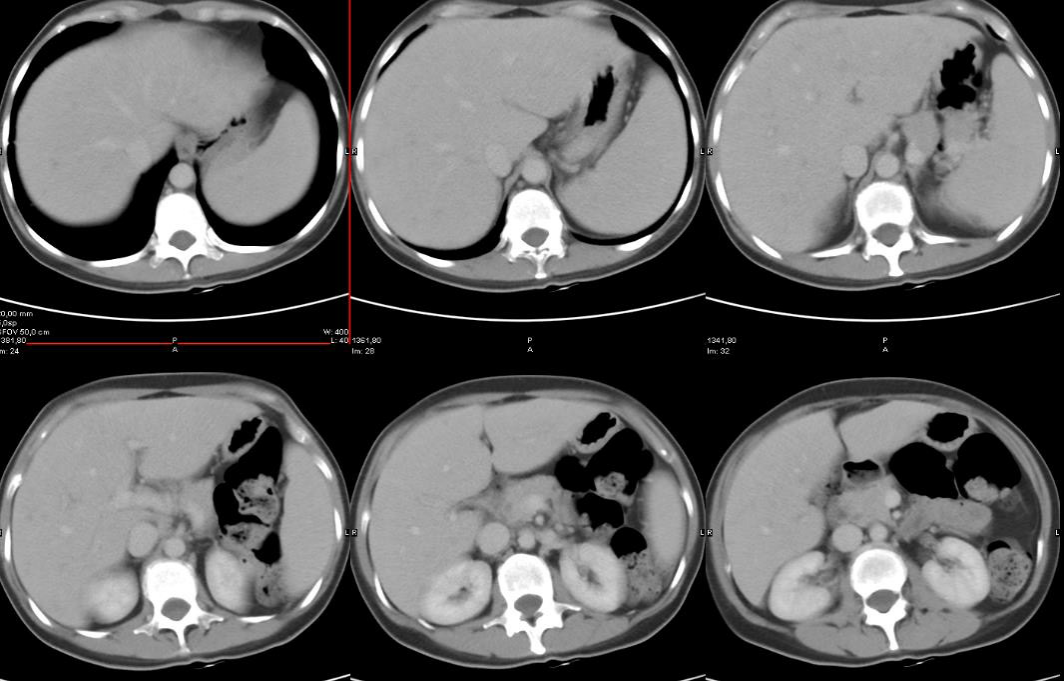
Anterior axial abdominal CT images (portal phase) show the presence of multiple hypodense nodules with a diameter between 5 mm and 1 mm, inhomogeneous enhancement, and ill-defined profiles

The patient’s glucose level was estimated at 94 mg/dL. ^18^F-FDG PET/CT scan showed multiple foci of intense FDG uptake in the liver; the largest uptake was reported in segment VI and the spleen (Figure 1). No other pathological uptakes were discovered in the rest of the body, particularly lymphatic tissues. The subsequent abdominal CT scan confirmed the presence of multiple hypodense hepatic and splenic lesions, resembling microabscesses (Figure 2).

**Figure 3:**
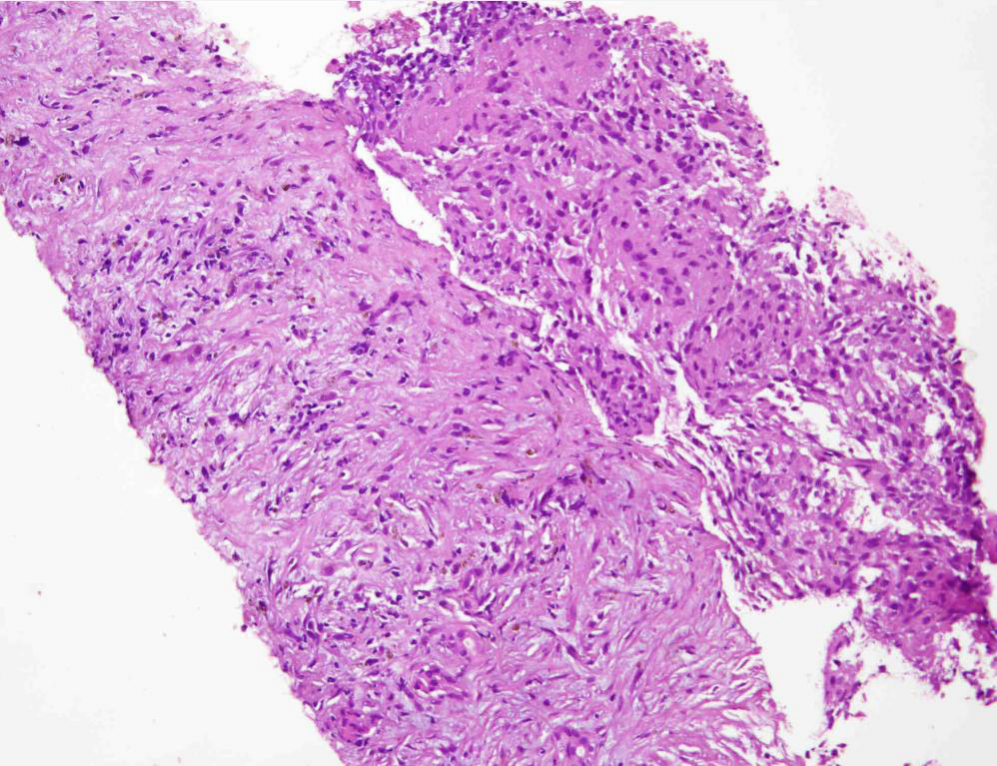
Liver biopsy of chronic disseminated candidiasis shows granuloma and necrotic septum on hematoxylin and eosin staining, with inflammatory infiltrate and bile ducts incorporated in fibrotic tissues

Hepatic biopsy of the largest lesion showed a chronic inflammatory process, similar to granuloma, with yeasts suggestive of hepatosplenic candidiasis (Figure 3). The patient was treated with antifungal therapy, using fluconazole. After 30 days, the fever disappeared and the subsequent abdominal CT scan yielded negative results.

## Discussion

Hepatosplenic candidiasis, also known as chronic disseminated candidiasis, often affects patients with hematologic malignancies, such as leukemia (1). Although the pathogenesis of this infection is unclear, invasion of Candida species from the gastrointestinal tract into the bloodstream may be a contributing mechanism (2).

The liver is the most commonly involved organ in hepatosplenic candidiasis, since the portal system receives the largest inoculum. The most common symptom of hepatosplenic candidiasis is persistent fever (3, 4). CT scan, ultrasonography, and magnetic resonance imaging (MRI) can reveal multiple characteristic lesions, resembling microabscesses in the liver, spleen, and sometimes other organs (5-7).

Despite high costs, MRI is the most sensitive technique for accurate diagnosis of hepatosplenic candidiasis, while CT scan and ultrasonography have lower sensitivities (8). A definitive diagnosis can be only established via biopsy, although this modality cannot be often performed due to the patient’s clinical condition. The treatment for chronic disseminated candidiasis is antifungal therapy by fluconazole (9).

The actual role of ^18^F-FDG PET/CT scan in evaluating hepatosplenic candidiasis remains unclear. In the literature, only few reports have shown FDG uptake in hepatosplenic candidiasis, and the utility of FDG PET/CT scan in selecting the best antifungal therapy (10-13). In this regard, Hot et al. (14) demonstrated that ^18^F-FDG PET/CT scan can be considered as a sensitive tool for the staging of invasive non-central nervous system fungal infections, such as chronic disseminated candidiasis.

The present case is of interest since it describes the potential role of ^18^F-FDG PET/CT scan in diagnosing hepatosplenic candidiasis, guiding the subsequent patient management, and helping determine the best site (lesion) for biopsy in particular.

This case report focused on hepatosplenic candidiasis in a patient with leukemia. Overall, positive ^18^F-FDG PET/CT scan after treatment may suggest an infective-inflammatory disease, e.g., hepatosplenic candidiasis, secondary to chemotherapy. Moreover, ^18^F-FDG PET/CT scan, unlike MRI, which investigates limited body segments, allows whole body imaging, which may detect any uptake throughout the body.
